# Supplementary material for: Adherence to Prescribed Acamprosate in Alcohol Dependence and 1-Year Morbidities and Mortality: Utilizing a Data Linkage Methodology
Source: J Clin Med. 2021 May 13;10(10):2102. doi: 10.3390/jcm10102102 (PMC8153116; doi:10.3390/jcm10102102)
Supplement: Supplementary file 1 [file jcm-10-02102-s001.zip › jcm-1184031-supplementary.pdf]

Supplementary Table 1. Descriptive statistics of biochemistry at the baseline when Acamprosate® was initiated

Predictor	Reference range	Mean (SD)	*p*-value
ALT	F: ≤ 34 IU/L<br>M: ≤ 45 IU/L	45 (2.9)<br>57 (3.4)	0.125
AST	F: 6-34 IU/L<br>M: 8-40 IU/L	48 (1.2)<br>57 (3.5)	0.634
GGT	F: 5-55 IU/L<br>M: 15-85 IU/L	78 (6.5)<br>97 (2.4)	0.769

ALT=ALanine aminotransferase; AST=ASpartate aminotransferase; γGT=Gamma-Glutamyl Transferase; SD= Standard Deviation; *p*= <0.05

Supplementary Table 2. Characteristics of individuals who died within 1 year of starting Acamprosate®.

	**Total number and characteristics of individuals prescribed Acamprosate®**	**Individuals prescribed Acamprosate® and died within one year of the study**
**Number**	3319	252
**Mean Age (SD)**	48.41 (11.8)	49.93 (12.1)
**Males**	2047 (61.0%)	179 (71.0%)
**Females**	1272 (39.0%)	72 (28.6%)
**SIMD**<br>**1**<br>**2**<br>**3**<br>**4**<br>**5**	 529 (16.0%)<br>471 (14.0%)<br>430 (12.6%)<br>374 (10.6%)<br>237 (8.0%)<br>1176 (35.5%)	 88 (34.9%)<br>65 (25.8%)<br>41 (16.3%)<br>23 (11.6%)<br>28 (6.0%)
**Adherence**<br>**Low**<br>**Good**	 2732 (87.0%)<br>587 (13.0%)	 217 (86.2%)<br>35 (13.8%)
**Alcohol**<br>**Direct**<br>**Not Direct**	 198 (31.0%)<br>323 (69.0%)	 101 (40.8%)<br>151 (59.2%)

SD= Standard Deviation; SUIMD= Scottish Index of Multiple Deprivation

Supplementary Table 3. Multivariate association between covariates and all cause mortality.

**Predictor**	**HR**	**95% CI**	***p*-value**
Age	1.15	1.05-1.27	***<0.001***
Sex			0.001
M	0.082	0.013-0.53	0.027
F	0.104	0.02-0.67	0.046
SIMD	1.125	1.02-1.24	<0.05
Low Adherence	1.2	1.03-1.4	***<0.001***
Due to Alcohol	0.99	0.86-1.2	0.957
Number of other medications			
0	1.0		
1-2	0.75	0.46-1.23	n.s.
3-5	0.56	0.22-1.22	n.s.
6+	0.44	0.16-1.18	n.s.

n.s.= not significant; M=Male; F=Female; SIMD= Scottish Index of Multiple Deprivation
HR=Hazard Ratio; CI= Confidence Interval
